# Population Genetics of an Ecosystem-Defining Reef Coral *Pocillopora damicornis* in the Tropical Eastern Pacific

**DOI:** 10.1371/journal.pone.0021200

**Published:** 2011-08-09

**Authors:** David J. Combosch, Steven V. Vollmer

**Affiliations:** Marine Science Center, Northeastern University, Nahant, Massachusetts, United States of America; Rutgers University, United States of America

## Abstract

**Background:**

Coral reefs in the Tropical Eastern Pacific (TEP) are amongst the most peripheral and geographically isolated in the world. This isolation has shaped the biology of TEP organisms and lead to the formation of numerous endemic species. For example, the coral *Pocillopora damicornis* is a minor reef-builder elsewhere in the Indo-West Pacific, but is the dominant reef-building coral in the TEP, where it forms large, mono-specific stands, covering many hectares of reef. Moreover, TEP *P. damicornis* reproduces by broadcast spawning, while it broods mostly parthenogenetic larvae throughout the rest of the Indo-West Pacific. Population genetic surveys for *P. damicornis* from across its Indo-Pacific range indicate that gene flow (i.e. larval dispersal) is generally limited over hundreds of kilometers or less. Little is known about the population genetic structure and the dispersal potential of *P. damicornis* in the TEP.

**Methodology:**

Using multilocus microsatellite data, we analyzed the population structure of TEP *P. damicornis* among and within nine reefs and test for significant genetic structure across three geographically and ecologically distinct regions in Panama.

**Principal Findings/Conclusions:**

We detected significant levels of population genetic structure (global R_ST_ = 0.162), indicating restricted gene flow (i.e. larvae dispersal), both among the three regions (R_RT_ = 0.081) as well as within regions (R_SR_ = 0.089). Limited gene flow across a distinct environmental cline, like the regional upwelling gradient in Panama, indicates a significant potential for differential adaptation and population differentiation. Individual reefs were characterized by unexpectedly high genet diversity (avg. 94%), relatively high inbreeding coefficients (global F_IS_ = 0.183), and localized spatial genetic structure among individuals (i.e. unique genets) over 10 m intervals. These findings suggest that gene flow is limited in TEP *P. damicornis* populations, particularly among regions, but even over meter scales within populations.

## Introduction

Populations at the periphery of species' distribution ranges often exist in suboptimal and/or unstable conditions at the organism's physiological or ecological limits [1]. They tend to be small as well as geographically and genetically isolated from central core populations [2], [3]. Their small population sizes and genetic isolation increase genetic drift and inbreeding and limit genetic diversity and cohesiveness among populations [4], [5], [6]. As a result, peripheral populations tend to have reduced genetic diversity and are often heavily reliant on local sources of recruitment rather than long-distance recruitment from core populations [e.g. 7,8,9,10]. Their isolation and low levels of genetic diversity may also hamper their ability to cope with environmental changes (e.g. global climate change) and to recover from disturbances, making them more vulnerable to extinction [11], [12]. However, small population sizes in genetic isolation can also facilitate local selection and adaptation, give rise to evolutionary innovation [13] and allow peripheral populations to exploit new ecological niches [14], [15].

One of the most isolated and peripheral biogeographic regions of the world's oceans is the Tropical Eastern Pacific (TEP). It is separated from the Indo-West Pacific by 5000 km of open-ocean that Darwin [16] described as “impassable” for shallow water marine species [17], [18]. While not completely impassable, the marine fauna of the TEP consists of a limited subset of trans-Pacific species (i.e. species distributed across the Pacific), which are typically interconnected by low levels of gene flow [e.g. 19,20,21,22,23,24,25], as well as numerous endemic species [22], [26], [27]. For example, 34 species of reef-building corals are recognized in the TEP (compared to 581 in the Western Pacific), of which 27 are trans-Pacific (80%) and seven (20%) are regional endemics [28]. Major environmental challenges for reef corals in the TEP include significant freshwater discharges and seasonal upwelling, leading to high turbidity, elevated nutrient levels and reduced seawater temperatures. Reef habitats in the TEP are confined mostly to a narrow continental shelf and often fragmented by long stretches of estuaries, mangroves and sandy beaches [27], [29].

The isolation of the TEP from the Indo-West Pacific and major differences in environmental factors and species compositions between these regions enabled significant ecological and life-history shifts in many TEP species. For example, the scleractinian coral *Pocillopora damicornis* is a minor reef-building coral in the Indo-West Pacific, but the primary reef-builder in the TEP [30], [31], [32]. *Pocillopora damicornis* in the TEP forms large, mono-specific carpets measuring tens to hundreds of hectares [33], that are thought to be maintained predominantly by asexual (i.e. vegetative) fragmentation [34], [35]. Moreover, while Indo-West Pacific *P. damicornis* populations brood mostly asexual (parthenogenetic) larvae [36], [37], populations in the TEP reproduce by broadcast-spawning its gametes [38]. This reproductive shift could have important genetic consequences since a broadcast spawning coral should not be able to produce parthenogenic larvae [39], [40] and because broadcast spawning corals are often considered to have higher dispersal potentials than brooding corals [e.g. 41,42,43,44,45], particularly over small spatial scales [46], [47], [48], [49], but see [50], [51]. Hermaphroditic broadcast-spawning corals might also have a higher potential for selfing and inbreeding resulting from external fertilization [52], [53], [54].

Population genetic surveys among larvae-brooding *P. damicornis* populations across the Indo-West Pacific indicate that larval dispersal (i.e. gene flow) is generally limited over hundreds of kilometers [e.g. 55,56,57] and in some cases even among neighboring reefs [e.g. 57,58,59]. Some of the highest levels of genetic structure have been described at the margins of *P. damicornis* Indo-Pacific range [55], [56], [57], [60]. For example, populations in South-East Australia are more genetically distinct over 700 km [FST = 0.32; 56] than more central Great Barrier Reef populations over 1200 km [FST = 0.06; 61]. Most studied populations of *P. damicornis* in the Indo-West Pacific also showed significant levels of local inbreeding and contained some level of clonality [61], [62], [63], [64], [65].

Little is known about the population structure of broadcast-spawning *P. damicornis* populations in the TEP. Combosch et al. [21] documented evidence for restricted transpacific dispersal between Central and Eastern Pacific populations of *P. damicornis,* using ribosomal DNA sequence data (F_ST_ = 0.419, p<0.001). Using multilocus genetic data, Pinzon & LaJeunesse [66] examined the genetic structure among multiple TEP *Pocillopora* species, including 7 well-recognized Indo-Pacific species (*P. damicornis*, *P. eydouxi*, *P. meandrina*, *P. verrucosa*, *P. capitata*, *P. elegans*, *P. woodjonesi*). They identified three genetic clusters or types (Type I, II & III) in their multi-species TEP sample of *Pocillopora*. All three of these types were found in *P. damicornis* from across the TEP. Type I was found throughout the TEP, including Panama, Type II was found only in Clipperton Atoll and Type III was found in Panama and Galapagos. Yet, no genetic structure was detected within the broadly distributed Type I group over 3500 km of the TEP.

One of the best-studied areas in the TEP is the Pacific coast of Panama, which is dominated by two major gulfs, the Gulf of Chiriqui in the west and the Gulf of Panama in the east. The two gulfs differ in several important ecological factors [27], [67], [68], [69], [70], [71], [72], most notably in the strength of seasonal upwelling, which is strong in the Gulf of Panama, but virtually absent in the Gulf of Chiriqui [27], [73]. The Azuero Peninsula, which separates the two gulfs, is a transition zone with moderate upwelling [74]. Corals from the two gulfs differ significantly in their thermal tolerances [74], [75] and ability to recover from major disturbances like El-Niño induced bleaching events [76].

**Figure 1:**
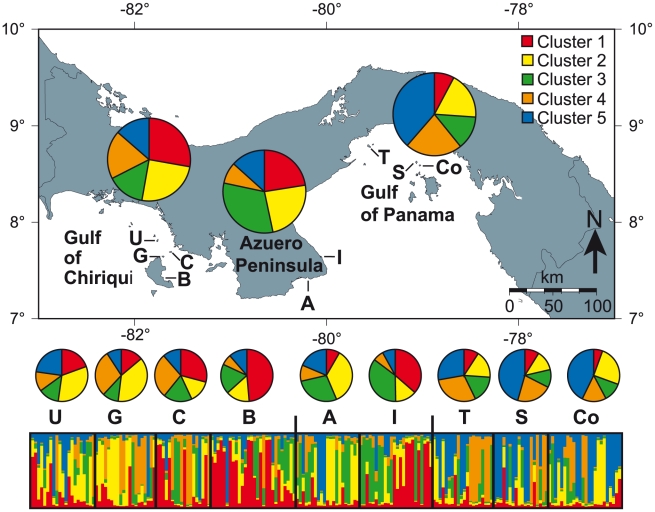
Map of Panama, showing the three main Pacific regions and the sampled locations. The pie charts show the distribution of the five genetic clusters identified by STRUCTURE 2.3 [141] in the three regional and nine reef populations. The bar plot shows each individual sample as a vertical bar with colors indicating the relative proportions of each genetic cluster. Map created on www.aquarius.ifm-geomar.de. U = Uva Island; C = Canal de Afuera Island; G = Granito de Oro Island; B  =  Bahia de Damas, Coiba Island; A  =  Achotines Bay; I  =  Iguana Island; S  =  Saboga Island; Co  =  Contadora Island; T  =  Taboga Island.

Using multilocus microsatellite data, we examined the population genetic structure of TEP *P. damicornis* within and among nine Panamanian populations across the three geographically and ecologically distinct regions (Figure 1). Based on the strong geographic and ecologic differences between the three regions, we predicted significant regional differences in *P. damicornis* population genetic structure. Within reefs, we analyzed spatial patterns of genetic diversity to estimate the relative contributions of clones versus unique genets to localized spatial genetic structure (SGS). We predict that high rates of vegetative fragmentation should lead to significant SGS due to clonal aggregations. In contrast, high rates of sexual reproduction by broadcast spawning should inhibit SGS due to increased outcrossing and the mixing of genotypes at the sea surface during larval development.

## Results

### Genetic diversity, heterozygosity and clonality

Multilocus microsatellite data (n≥5 loci) was obtained for 207 *Pocillopora damicornis* colonies from nine reef populations (Tables 1 and 2). The six amplified loci yielded between four and ten alleles each (mean N_A_ = 7). Three pairs of loci showed indications of significant linkage disequilibria, but none showed consistent significant linkage disequilibria in more than two populations. Consequently, all six loci were considered as unlinked. Significant heterozygote deficits were detected in five loci (Table 1), but only Pv2 showed significant deficits in all populations, which is indicative of null alleles. The program Micro-Checker confirmed the presence of null alleles in Pv2, which was then excluded from further analyses.

**Table 1:** Population genetic indices for the six microsatellite loci.

Marker	N	N_A_	Ho ± SE	He ± SE	F_IS_
** Pd2**	183	8	0.551±0.035	0.633±0.019	0.239**
** Pd4**	161	5	0.292±0.056	0.489±0.044	0.606**
** Pd5**	188	9	0.509±0.031	0.641±0.014	0.267**
** Pd6**	187	6	0.625±0.077	0.629±0.021	0.077
** Pv2**	173	4	0.109±0.033	0.426±0.062	0.831**
** Pv6**	172	10	0.597±0.057	0.738±0.020	0.298**
**Total**	194	42	0.447±0.032	0.593±0.020	0.367**

Indices are based on genets, i.e. unique genotypes, per locus and over all loci ( =  Total).

N  =  Number of samples; N_A_  =  Number of alleles per locus; Ho  =  Heterozygosity observed; He  =  Heterozygosity expected; F_IS_  =  (He-Ho)/He  =  Inbreeding coefficient; SE  =  Standard Error; *p<0.05, **p<0.005 per loci

**Table 2:** Population genetic indices for the nine populations.

Region	Pop	N	N_G_/N	N_A_ ± SE	Ho ± SE	He ± SE	F_IS_ ± SE
** Gulf of**	**U**	23	0.91	5.2±0.8	0.589±0.069	0.624±0.049	0.062±0.065
**Chiriqui**	**G**	21	0.95	5.4±0.9	0.531±0.110	0.687±0.018	0.236±0.154*
	**C**	18	1.00	5.0±0.9	0.378±0.081	0.640±0.039	0.421±0.103*
	**B**	30	0.93	4.8±0.5	0.580±0.081	0.613±0.075	0.065±0.026
**Azurero**	**A**	23	0.91	4.4±0.7	0.615±0.079	0.607±0.044	−0.048±0.168
**Peninsula**	**I**	24	1.00	4.6±1.0	0.544±0.120	0.605±0.080	0.143±0.159*
** Gulf of**	**S**	22	0.82	4.0±0.3	0.390±0.072	0.575±0.059	0.336±0.087*
**Panama**	**Co**	25	0.96	4.6±0.7	0.445±0.093	0.648±0.047	0.314±0.128*
	**T**	21	0.95	4.2±0.4	0.562±0.057	0.634±0.019	0.114±0.084*
** Total**		207	0.94	4.7±0.2	0.515±0.029	0.626±0.016	0.183±0.042*

Indices are based on genets, i.e. unique genotypes, except for number of samples.

Pop  =  Population: U  =  Uva, G  =  Granito, C  =  Canal de Afuera, B  =  Bahia de Damas, A  =  Achotines, I  =  Iguana, S  =  Saboga, Co  =  Contadora, T  =  Taboga;

N  =  Number of samples; N_G_  =  Number of unique genotypes; N_G_/N  =  Genet ration; N_A_  =  Average number of alleles per population; SE  =  Standard Error; Ho  =  Heterozygosity observed; He  =  Heterozygosity expected; F_IS_  =  (He-Ho)/He  =  Inbreeding coefficient; *p<0.005 per pop

Using the five remaining loci, 194 unique genets were identified with a high probability of identity (p<3×10^−5^; Table 2) among the 207 genotyped samples. All nine populations were comprised predominantly of unique, i.e. sexually derived genets (93.8±5.5%). Clonal genotypes were detected in all but two populations (Canal & Iguana), but only Saboga (Gulf of Panama) contained more than two clones (n = 4, i.e. 18%). No clonal genotypes were shared among populations, in contrast to larvae-brooding Indo-West Pacific populations [64], [77]. Since clones introduce biases into population genetic analyses, only unique genets were used in subsequent analyses, unless otherwise noted.

Using the genet only dataset, significant heterozygote deficits were detected in six out of the nine populations (Table 2), indicating widespread local inbreeding. Inbreeding was significant in the Gulf of Panama (F_IS_ = 0.255±0.122) and in the Gulf of Chiriqui (F_IS_ = 0.196±0.171), but not in the Azuero Peninsula (F_IS_ = 0.048±0.135) due to an excess of heterozygotes in Achotines. Each population contained on average 4.7 alleles per locus. Slightly more alleles were detected in the Gulf of Chiriqui (avg. N_A_ = 5.1±0.26) than in the Azuero Peninsula (4.5±0.20) and in the Gulf of Panama (4.3±0.31). Interestingly, private alleles were only found in Gulf of Chiriqui populations, which contained the only regional-specific allele (Pd2, 223 bp) as well.

### Genetic Structure among reefs and regions

Hierarchical AMOVA revealed significant levels of population genetic structure (p<0.005) among populations, among regions, and among populations within regions (Table 3). Genetic structure among populations was highly significant for both global R_ST_ (0.162; p<0.001) and global F_ST_ (0.053; p<0.001). For the R-statistics, population structure was equivalent among regions (R_RT_ = 0.081) and among populations within regions (R_SR_ = 0.089). All three regions were significantly different from each other (p<0.001), but the two gulf regions were most distinct (pairwise R_ST_ = 0.138), whereas the Azuero Peninsula, which lies between the two gulfs, showed lower pairwise genetic structure (Gulf of Chiriqui R_ST_ = 0.070; Gulf of Panama R_ST_ = 0.101). For the F-statistics, population structure was predominantly due to differences among populations within regions (F_SR_ = 0.045; Table 3).

**Table 3:** Hierarchical AMOVA results showing levels of genetic structure among regions (R_RT_/F_RT_), among populations within regions (R_SR_/F_SR_) and among populations (R_ST_/F_ST_).

Source of Variation	df	Variance	Variation	Differentiation	p
**R-statistics**					
Among Regions	2	1331	8.1%	R_RT_ = 0.081	<0.001
Among Populations	6	1336	8.1%	R_SR_ = 0.089	<0.001
Within Populations	379	13754	83.8%	R_ST_ = 0.162	<0.001
**F-statistics**					
Among Regions	2	0.014	0.8%	F_RT_ = 0.008	0.004
Among Populations	6	0.081	4.5%	F_SR_ = 0.045	<0.001
Within Populations	379	1.700	94.7%	F_ST_ = 0.053	<0.001

**Figure 2:**
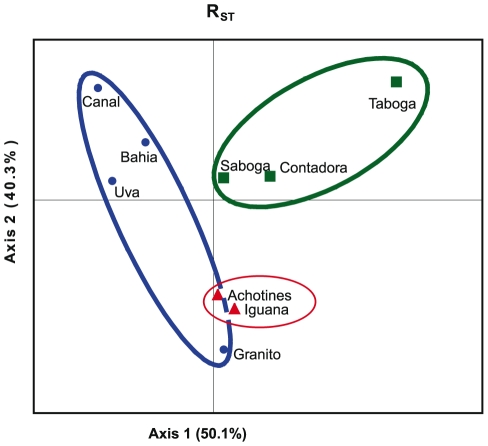
Principal component analysis of *Pocillopora damicornis* populations, constructed using GenAlEx 6.1 [**130**]. Distances between populations were analyzed as RST pairwise genetic distances. The two axes explained 90.4% of the total variation. Blue circles  =  Populations in the Gulf of Chiriqui; Red triangles  =  Azuero Peninsula; Green squares  =  Gulf of Panama.

Fourteen out of 36 pairwise R_ST_ comparisons between populations were significant after sequential Bonferroni adjustments (Table 4). Twelve of the significant pairwise R_ST_s were between populations from different regions, whereas only two comparisons within regions were significant, both involving the Gulf of Chiriqui population Granito. These strong regional differences can be seen as well in the principal coordinate analyses, where populations cluster into distinct regional groupings (Figure 2). However, no significant isolation-by-distance pattern was observed among populations (p = 0.013).

**Table 4:** Pairwise R_ST_ (above diagonal) and F_ST_ (below diagonal) between TEP *P. damicornis* populations.

		Gulf of Chiriqui				Azuero Peninsula		Gulf of Panama		
		U	G	C	B	A	I	S	Co	T
**Gulf of**	**U**		0.114*	0.025	0.025	0.086*	0.092*	0.053*	**0.102** *	**0.309** *
**Chiriqui**	**G**	0.035*		0.033*	**0.124** *	0.000	0.000	0.065*	0.085*	**0.281** *
	**C**	0.033*	**0.229** *		0.029	**0.156** *	**0.188** *	0.080*	**0.129** *	**0.316** *
	**B**	**0.065** *	**0.073** *	0.047*		**0.086** *	**0.116** *	0.014	0.065*	**0.243** *
**Azuero**	**A**	0.015	0.001	0.037*	**0.069** *		0.013*	0.046*	0.072*	**0.222** *
**Peninsula**	**I**	0.039*	**0.048** *	0.025*	**0.045** *	0.029*		0.039*	0.037*	**0.227** *
**Gulf of**	**S**	0.019	**0.061** *	0.015	**0.053** *	0.040*	0.022*		0.000	0.133*
**Panama**	**Co**	0.025*	**0.039** *	**0.040** *	**0.060** *	0.031*	**0.033** *	0.000		0.091*
	**T**	**0.087** *	**0.072** *	**0.064** *	**0.065** *	**0.071** *	**0.069** *	**0.046** *	0.030*	

*p<0.05; **bold**  =  significant after Sequential Bonferroni correction

GC  =  Gulf of Chiriqui, AP  =  Azuero Peninsula, GP  =  Gulf of Panama; U  =  Uva, G  =  Granito, C  =  Canal de Afuera, B  =  Bahia de Damas, A  =  Achotines, I  =  Iguana, S  =  Saboga, Co  =  Contadora, T  =  Taboga;

Bayesian clustering implemented in STRUCTURE detected five genetic clusters in the dataset (Figure S1). All five clusters were found in each population, but their distribution differed significantly among populations and particularly among regions (Figure 1). The genetic clusters 1 and 2 were most common in the Gulf of Chiriqui (28% & 25%, respectively), clusters 4 and 5 were most prevalent in the Gulf of Panama (22% & 39%) and cluster 3 was dominant in the Azuero Peninsula (32%). Cluster 1 and 2 were also common in the Azuero Peninsula (23% and 24%), which indicates that its *P. damicornis* populations are more similar to the Gulf of Chiriqui than to the Gulf of Panama - as indicated by the pairwise R_ST_ values.

### Fine-scale spatial genetic structure (SGS) on reefs

**Figure 3:**
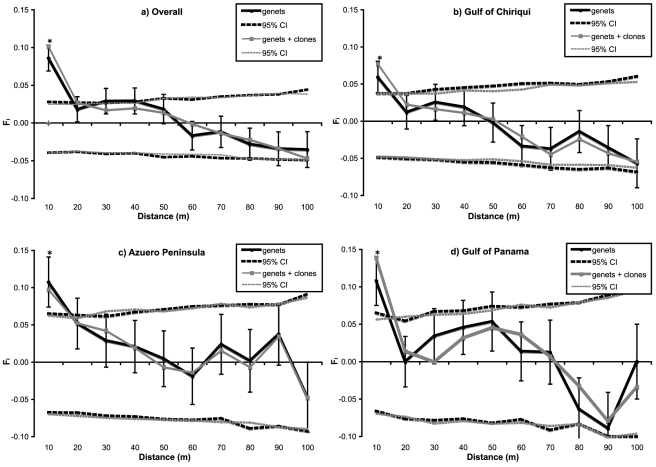
Spatial correlograms of the SpaGeDi [**145**] analysis of Spatial Genetic Structure (SGS) among specimen within discrete distance classes. Black lines represent the genet dataset, excluding clones and grey lines show the results for the ramet dataset, including clones. Dashed lines are the permuted 95% confidence intervals. Error bars are 95% bootstrap errors of the genet datasets. **Figure 3a**) Spatial correlogram of the average pairwise kinship F_ij_ [146] as a function of distance over all populations. **Figure 3b**–**d**) Spatial correlograms over the populations in each of the three regions, b) the Gulf of Chiriqui, c) the Azuero Peninsula and d) the Gulf of Panama.

In addition to the genetic structure among regions and populations, strong fine-scale spatial genetic structure (SGS) was detected among individuals within reefs. SGS was significant over 10 m intervals in both the ramet dataset (i.e. including clones; F_ramets_ = 0.102; p<0.001) and the genet dataset (i.e. excluding clones; F_genets_ = 0.086, p<0.001). This indicates that corals 10 meters apart are significantly more closely related to each other than to the rest of the population. Kinship among individuals (F_ij_) was only slightly elevated when clones were included, reflecting the low frequency of clones in the dataset (Figure 3). While SGS is to be expected in coral populations due to clonal aggregations as a consequence of asexual (vegetative) fragmentation, significant SGS in the genet dataset indicates that the observed structure is due to non-random spatial relatedness among distinct, i.e. sexually produced, genets. Across all nine populations, the average genetic patch size (i.e. the distance at which individuals on average are as related as across the entire population) was 50 m and the Sp statistic was 0.055 [i.e. rather high compared to plant literature data; 78].

Significant levels of SGS out to 10 m were detected in all three regions (p<0.05; Figure 3b–d). Average F_10m_-kinship in the Azuero Peninsula and Gulf of Panama was similarly high (F_10m_ ∼0.109), almost equivalent to a first-cousin relatedness (F_IJ_ = 0.125) whereas F_10m_-kinship in the Gulf of Chiriqui was two times lower (0.059; p<0.05). The genetic patch size was almost twice as large in the Gulf of Panama (70 m) than in the Gulf of Chiriqui and Azuero Peninsula (40 m). These differences in the strength and extent of SGS are reflected in the Sp statistic, which indicates that SGS is strongest in the western Gulf of Panama (0.062), intermediate in the central Azuero Peninsula (0.059) and lowest in the eastern Gulf of Chiriqui (0.049).

## Discussion

The Tropical Eastern Pacific (TEP) is one of the most geographically isolated biogeographic regions in the world's oceans [16]. Its isolation has profound consequences for the diversity and ecology of TEP ecosystems, most notably coral reefs [27]. TEP coral reefs are remarkable since they are built predominantly by *Pocillopora damicornis* [30], [31], [32]. TEP *P. damicornis* also distinguish by its broadcast gamete-spawning reproductive strategy. Multilocus microsatellite data from nine Panamanian populations demonstrate that TEP *P. damicornis* populations from Panama are characterized by strong population genetic structure (R_ST_ = 0.162), within and particularly among its three major regions, the Gulf of Chiriqui, the Azuero Peninsula and the Gulf of Panama. Moreover, the large and mono-specific stands of *P. damicornis* that characterize TEP reefs are not dominated by a few, highly clonal genotypes as previously thought [34], [35], but instead are comprised of numerous distinct genets, indicating frequent sexual reproduction. Significant fine-scale spatial genetic structure (SGS) among individual genets and highly significant levels of heterozygote deficits further indicate widespread inbreeding and limited gene dispersal over meter scales, which is unusual for free-spawners. These tiered layers of significant population genetic structure indicate that gene flow (i.e. larval dispersal) is restricted among and within reefs, particularly between the environmentally distinct regions, which indicates a significant potential for regional adaptations to the different ecological conditions in each region.

### Population Structure

As hypothesized, we detected strong genetic differences among the three geographically and ecologically distinct regions in Panamanian *P. damicornis* populations (R_RT_ = 0.081). Panama's two major gulfs – the Gulf of Chiriqui and the Gulf of Panama - which are geographically most distant, were most genetically distinct (R_ST_ = 0.138). These genetic differences correspond with pronounced ecological differences between these two regions, e.g. in species compositions and environmental factors [e.g. 38,67,73]. The most significant environmental difference is the pattern of seasonal upwelling, which is strong in the Gulf of Panama dropping temperatures down to 15°C, but is virtually absent in the Gulf of Chiriqui [27], [73]. The Azuero Peninsula, which bisects the two regions, is a transition zone with intermediate upwelling conditions [74]. It also has intermediate levels of genetic structure compared to the other regions (R_ST_ = 0.070 & 0.101).

Upwellings deliver cool, nutrient-rich waters to the near-shore environment. In tropical habitats like the TEP, they have significant impacts on nutrient and temperature-sensitive reef fauna [68], [69]. In the TEP, variations in the severity of upwelling restrict the regional and local distributions of coral reefs [27], [70] and of major reef taxa, including corals [27] and key corallivores like the sea star *Acanthaster planci* [67]. Lower temperatures associated with seasonal upwellings reduce reef development [71], coral growth rates [68] and coral reproduction [38], while elevated nutrients increase competition with algae [70], [79], bioerosion [72] and water turbidity [27]. Corals from regions with different upwelling regimes often display different thermal tolerances, indicating local adaptations. For example, *P. damicornis* from the non-upwelling Gulf of Chiriqui are less sensitive to higher temperatures than those from the upwelling Gulf of Panama [74], [75]. Similar differences in thermal adaptation among *P. damicornis* populations have also been reported from the Great Barrier Reef [80]. Yet, upwellings can also buffer against critically high sea surface temperatures and reduce coral bleaching and mortality. For example, during the 1997/98 El-Niño, seasonal upwelling in the Gulf of Panama reduced rising seawater temperatures (mean 27°C), preventing significant coral bleaching and mortality. In contrast, at the same time in the non-upwelling Gulf of Chiriqui, mean seawater temperature rose to 30°C causing severe coral bleaching and mortality [76]. While reefs in upwelling environments may thus be better able to weather predicted increases in sea surface temperatures and El Niño frequencies resulting from global climate change [e.g. 81], coral populations in non-upwelling habitats seem to be better adapted to elevated temperatures [74], [75]. The genetic structure of Panamanian *P. damicornis* indicates that even among broadcast-spawning coral populations, adaptations to different thermal regimes can occur over distances of ∼100 km.

Limited inter-regional gene flow in Panamanian *P. damicornis* may also explain prolonged differences in post-disturbance reef recovery between the Gulf of Chiriqui and the Gulf of Panama. For example, the 1982/83 El Niño caused mass coral mortalities and reduced average coral cover on most Panamanian reefs by 80%, but even though mortality rates were similar in both gulfs, reefs in the Gulf of Panama had recovered by 1992, whereas reefs in the Gulf of Chiriqui recovered only 3–6% of their populations by 1997 [76]. The significant population genetic structure between regions indicates that larval dispersal from recovered Gulf of Panama populations to the still depauperate Gulf of Chiriqui populations was very limited and this may have prolonged the recovery of reefs in the Gulf of Chiriqui. Moreover, due to the limited gene flow (i.e. larval dispersal) between individual reefs, damaged populations depended strongly on surviving colonies for repopulation. *Acanthaster* predation on surviving colonies in the Gulf of Chiriqui potentially hampered initial recovery significantly, while corallivorous sea stars are absent in the Gulf of Panama. Another important factor for the differences in *P. damicornis* recovery between Gulfs may be geographic differences in *Symbiodinium* identity (or type). Baker et al [82] showed that in the Gulf of Panama, *Pocillopora* corals with clade C bleached severely in 1997 while colonies with clade D were unaffected by bleaching, which lead to a relative increase in colonies with clade D from 43% in 1995 to 63% in 2001. Higher levels of clonality in the Gulf of Panama (e.g. Saboga) further indicate that asexual fragmentation might have played a more prominent role in the recovery of Gulf of Panama populations compared to their Gulf of Chiriqui counterparts.

Using multiple genetic markers, Pinzon & LaJeunesse [66] recently suggested that TEP *Pocillopora* are comprised of three distinct genetic types (Type I–III), independent of species identity and morphology. This is controversial because they included at least seven well-recognized Indo-Pacific species that must then either be reclassified or synonymized by their genetic types. Their dataset included a limited number of *P. damicornis* samples from the Gulf of Panama (n = 26), which were comprised of two of the three genetic types (Type I & II). Using a similar set of microsatellites, our STRUCTURE analyses do not support two distinct genetic types, but instead identified five genetic clusters (Figure 1 & Figure S1). In addition, Pinzon & LaJeunesse [66] failed to detect significant genetic structures within their *Pocillopora* type I over 3500 km from Mexico to Panama and the Galapagos. Given that we observe strong genetic structure in *P. damicornis* within Panama, it seems likely that the absence of geographic structure in Pinzon & LaJeunesse's [66] analyses are an artifact of pooling multiple species (i.e. gene pools) for population genetic analyses.

### Comparisons with brooding Indo-West Pacific *P. damicornis populations*

Similar levels of population genetic structure over scales of hundreds of kilometers have also been observed in other brooding and broadcast-spawning coral species in the Indo-Pacific and the Caribbean [83], [84]. In *P. damicornis*, allozyme studies from across the Indo-West Pacific indicate that populations exhibit a range of significant population genetic structures with F_ST_ values ranging from 0.06 to as high as 0.39 (Table 5). Some of the highest levels of population structure have been detected in isolated or peripheral populations. For example, F_ST_ values among high-latitude *P. damicornis* populations in south-east Australia averaged 0.37 over 700 km [56] compared to 0.06 among Great Barrier Reef populations over 1200 km [61]. Similarly, F_ST_ values among the southernmost *P. damicornis* populations around Lord Howe Island [57] were twice as high as among similarly spaced populations on the Great Barrier Reef (F_ST_ = 0.102 vs. F_ST_ = 0.044)[58].

**Table 5:** *Pocillopora damicornis* population genetic survey (sorted by F_ST_).

Locations	Scale (km)	F_ST_	F_IS_	Clones	Marker	Reference
GBR (One Tree Island)	5	0.04*	0.23	18%	Allozymes (7 loci)	Ayre & Miller 2004
GBR (One Tree Island)	1	0.05*	0.03-	13%	Allozymes (5 loci)	Benzie et al. 1995
GBR (One Tree Island)	5	0.06*	0.11*	17%	Allozymes (8 loci)	Sherman et al. 2006
GBR	1200	0.06*	0.28*	20%	Allozymes (7 loci)	Ayre et al. 1997
Japan (Okinawa)	650	0.06*	0.18*	39%	Allozymes (7 loci)	Adjeroud & Tsuchiya ‘99
Northwest Australia	900	0.08*	0.18	85%	Allozymes (6 loci)	Whitaker 2006
Lord Howe Island	10	0.10*	0.17	28%	Allozymes (7 loci)	Miller & Ayre 2004
Lord Howe Island to GBR	2500	0.15*	N/A	N/A	Allozymes (7 loci)	Ayre & Hughes 2004
Southeast Australia to GBR	1200	0.24*	0.44	49%	Allozymes (8 loci)	Miller & Ayre 2008b
Southeast Australia	700	0.32*	0.47	42%	Allozymes (8 loci)	Miller & Ayre 2008b
Southwest Australia	400	0.39*	1N/A	82%	Allozymes (4 loci)	Stoddart 1984
Hawaii (Kaneohe Bay)	10	N/A	N/A	71%	Allozymes (4 loci)	Stoddart 1986
East Africa	860	0.02*	0.26*	^2^ 13%	Microsats (6 loci)	Souter et al. 2009
Indonesia	3300	0.05	N/A	2%	Microsats (9 loci)	Starger et al. 2010
**Tropical Eastern Pacific**	** 400**	**0.05** *	**0.18** *	** 6%**	**Microsats (5 loci)**	**THIS STUDY**
Taiwan (Nanwan Bay)	0.5	N/A	N/A	66%	Microsats (7 loci)	Yeoh & Dai 2010
TEP to Central-West Pacific	8000	0.42*			ITS2–5.8S	Combosch et al. 2008

1 =  consistent heterozygote deficits, reported as differences between He & Ho, i.e. inconvertible; ^2^ =  two different types of *P. damicornis*;

* =  significant; -  =  not significant;

Direct comparisons of population genetic data are possible with two *P. damicornis* microsatellite datasets from peripheral populations in East-African [64] and core populations in the Indonesian Archipelago [77] since they used the same or similar subsets of the microsatellite loci used here. Both studies found highly significant population genetic structure. Levels of genetic differentiation among the peripheral East-African populations were lower (F_ST_ = 0.02 over 860 km) than among TEP populations (F_ST_ = 0.05 over 400 km). Indonesian populations show the same degree of genetic differentiation as TEP populations (F_ST_ = 0.05) over a 10-fold wider geographic distance (3000 km vs. 400 km). We observed a much lower allelic diversity in the TEP (N_A_/locus = 7) compared to *P. damicornis* in Indonesia and East-Africa (N_A_/locus = 14.0 & 13.4, respectively). Lower allelic diversities are expected for peripheral populations and the lower diversity in TEP populations, compared to the similarly peripheral East-African populations, is likely due to the extreme isolation of the TEP and very limited transpacific gene flow [FST = 0.419; 21]. As expected, inbreeding was found to be highly significant in both peripheral populations, in the TEP and in Eastern Africa [64]. Comparisons among *P. damicornis* allozyme studies confirm all three population genetic trends in peripheral populations: 1) higher genetic structure [e.g. 62 vs. 56], 2) decreased allelic diversity [56] and 3) higher inbreeding [56]. These patterns have also been observed in peripheral populations of other coral species [10], [55], [85], [86].

Our results indicate lower genetic diversity and higher levels of population structure among broadcast-spawning populations (TEP) than among larvae brooding populations (Indo-West Pacific), which may reflect intrinsic differences between the two reproductive strategies. In Indo-West Pacific populations, parthenogenetic larvae brooding preserves genotypes, which reduces the rate of genetic drift and allelic loss, while allelic diversity in TEP populations is likely reduced due to reproductive sweepstakes, promoted by its broadcast-spawning reproductive strategy [54], [87], [88]. Contrary to our results, inter-specific comparisons mostly indicate that brooding corals have reduced dispersal potentials compared to broadcast-spawners [42], [43], [45], [89]. This indicates that population structure in reef corals is not predominantly driven by reproductive strategies. Other factors are likely as important, for example effective population sizes and species-specific larval behaviors [e.g. 51,90].

### Fine-scale Population Genetic Structure

The observed fine-scale spatial genetic structure (SGS) and the high inbreeding coefficients (average F_IS_ = 0.18) indicate that gene dispersal is limited in Panamanian *P. damicornis*, not only between reefs, but within reefs as well. Fine-scale SGS arises from a variety of historic, demographic, and evolutionary processes [78], [91], [92]. Over small spatial scales, SGS is most often the result of limited gene dispersal and can be influenced by a broad array of life-history and reproductive traits including clonality, selfing, inbreeding, fertilization, fecundity, larval life history, and recruitment [78], [91], [93]. Clonality can be an important determinant of SGS because clonal aggregations increase the average kinship over short distance intervals [e.g. 46]. We observed fine-scale SGS out to 10 m in both, the genet-only and the genet-plus-clone dataset, but since clones were rare (6.2% of samples), clonality had only a small effect on the observed SGS (Figure 3). Instead, the fine-scale SGS observed in TEP *P. damicornis* was predominantly driven by significant non-random relatedness among individual genets out to 10 meters, which is unusual for free-spawning benthic marine populations.

The two most likely explanations for the observed SGS among genets in TEP *P. damicornis* are cohort recruitment (i.e. SGS among larval recruits, independent of parents) or proximity recruitment (i.e. SGS among parents and recruits). In brooding coral species, proximity recruitment is facilitated by the internal (i.e. stationary) larval development and the frequent settlement of larvae within meters of the maternal colony [94], [95], [96]. Although broadcast-spawning was never directly observed, several independent studies provide convincing evidence that TEP *P. damicornis* do not release brooded larvae [38], [97], [98] but instead free-spawn gametes [38]. In broadcast-spawning corals, like TEP *P. damicornis*, larval development occurs externally and larval settlement after gamete release takes at least 1–3 days [99]. Although corals generally spawn during times of minimal water movement [100], [101], it is unlikely that drifting coral larvae would stay so close together (cohort recruitment) or remain within meters of the maternal colony (inhibiting proximity recruitment) over several days during their dispersive phase to create such a fine-scale spatial genetic structure (10 m).

One potential scenario that could significantly reduce passive dispersal would be if gametes and/or larvae sink down and escape the flow of the water column. Although coral gametes typically float on the sea surface during fertilization and development [102], gametes of two *Pocillopora* [P. eydouxi & P. verrucosa, 103] and several other coral species (e.g. *Fungia scutaria*, *F. fungites*, *Goniastrea favulus*) are known to be negatively buoyant [40], [51], [104], [105]. Negatively buoyant gametes sink down after spawning and fertilize close to the substrate. Fertilization at the substrate also favors mating among nearby colonies, which limits gene dispersal, and should facilitate SGS. Even if fertilization occurs at the sea surface but the zygotes sink down promptly, larval dispersal would still be reduced considerably. A minority of propagules could then remain in the water column to account for long-distance dispersal. Whether TEP *P. damicornis* gametes or larvae are positively or negatively buoyant is currently unknown, since gamete spawning has not yet been observed directly.

A second indication for limited gene dispersal within populations is the significant inbreeding coefficients. Inbreeding is a consequence of non-random mating among a limited number of individuals, which limits gene dispersal and promotes SGS [106]. The broadcast-spawning reproductive strategy of TEP *P. damicornis* might facilitate inbreeding in several ways. Firstly, the external fertilization of broadcast-spawners lacks the potential parental control of internal fertilization in brooders [52], [53]. Secondly, the high fecundity of broadcast-spawners leads to reproductive sweepstakes, which can promote inbreeding [54], [87], [88]. However, heterozygote deficits were as pronounced in larvae-brooding East-African populations (F_IS_ = 0.26), and significant inbreeding is common in *P. damicornis* populations throughout the Indo-Pacific, regardless of reproductive strategy, local environment, population history, and analyzed genetic marker (Table 5). Inbreeding, and potentially SGS, may thus be inherent features of *P. damicornis*.

While SGS is common in plants [reviews by 78,107,108], it has only been documented in four other coral species. Underwood *et al.* [50] described a similar pattern of SGS in the brooding coral *Seriatopora hystrix*, including a significantly elevated kinship among genets in the smallest distance interval (F_1_ = 20 m) and a roughly comparable genetic patch size (80 m). Among broadcast-spawning corals, Stoddart [46] and Miller and Ayre [51] found no significant genet SGS in *Acropora digitifera* and *Goniastrea favulus,* respectively. However, Miller and Ayre [51] found significant genet SGS in the broadcast-spawning *Platygyra daedalea,* including significantly positive kinship in the smallest distance interval (F_1_ = 5 m) and a Genetic Patch Size of only 17 m. This is the only other documented broadcast-spawning coral with genet SGS. To account for this pattern, Miller and Ayre [51] suspected “an unknown element of larval behavior or development” - e.g. that larvae might be negatively buoyant.

Small-scale SGS over tens of meters has been documented for at least eight other sessile marine invertebrates, including two sponges [109], [110], a temperate soft coral [47], a black coral [48], a red coral [49], a bryozoan [111] and two tunicates [112], [113]. For six out of these eight taxa, SGS seems to be driven by limited dispersal of brooded, philopatric larvae. Both broadcast spawners with significant SGS, the Anthipatharian coral *Antipathes fiordensis* [48] and the solitary tunicate *Styela plicata* [113] have negatively buoyant larvae (*A. fiordensis*) or eggs (*S. plicata*).

### Clonal structure

Because of its large mono-specific stands and little to no detectable larvae recruitment [97], TEP *P. damicornis* reefs were long thought to be highly clonal, persisting locally through vegetative asexual fragmentation [34], [35]. Our data indicate that only 6% of corals sampled every 10 m apart were clones, i.e. big clones (>10 m) were uncommon. In contrast, 94% of samples consist of unique, sexually-derived genets. This indicates that sexual recruitment is sufficient to maintain high levels of genet diversity in TEP *P. damicornis*. Simulation models by Neigel & Avise [114] indicate that in populations of the Caribbean coral *Acropora cervicornis,* which depends heavily on vegetative fragmentation, 5% sexual recruitment (i.e. 95% asexual) is enough to generate high genotypic diversities (∼60%), independent of initial genet diversity.

Compared to other *P. damicornis* studies, levels of clonality in the TEP (6%) seem low. However, direct comparisons among studies are biased by different sampling strategies and genetic markers. Lower levels of clonality tend to be detected with microsatellites compared to allozyme markers (avg. 22% vs. 43%; Table 5) and with large sampling intervals compared to exhaustive samplings [e.g. 80,81 and the present study vs. 37]. Comparisons among studies using the same marker and similar sampling indicate higher levels of clonality in peripheral populations (TEP, East Africa & Lord Howe Island) compared to more central populations (Indonesia & Great Barrier Reef)(Table 5). Higher clonality in peripheral populations has been observed in other corals [10], [115] and terrestrial plants [7], [116]. Clonal reproduction is considered to enable persistence in the commonly adverse environmental conditions towards range margins, at the expense of sexual reproduction benefits [117]. One example of reduced sexual benefits due to asexual reproduction is limited gene flow among and within populations as described here for the peripheral TEP *P. damicornis* populations.

### Conclusion

Regional structure and localized SGS detected in Panamanian *Pocillopora damicornis* has important consequences for the conservation management of TEP coral reefs. Recent devastating mass mortalities, for example due to El Niño, demonstrate the fragility of TEP coral reefs [118], [119], [120], [121], [122]. However, regional populations reacted very differently to these large scale disturbances [76] and are further differently affected by regional disturbances including *Acanthaster* predation [123], [124], toxic algal blooms [125] and seasonal upwellings [70]. In combination with the significant genetic differences among regions, these variations highlight the importance of environmentally, ecologically and genetically defined regional management and conservation units in Panama and possibly throughout the TEP. Limited gene flow and significant genetic differences between reefs further indicate that local population dynamics on individual reefs are vital for reef resilience and adaptation in Panama. For example, although the limited gene flow potentially hampers population recovery, it also allows for local and regional adaptation, which should be preserved and accounted for in conservation management.

## Materials and Methods

Collection of coral samples for this project was approved by the Autoridad Nacional del Ambiente and was conducted under permit number ANAM SEA7108.

### Sampling Sites

*Pocillopora damicornis* was sampled from nine reefs out of three regions (Gulf of Chiriqui, Azuero Peninsula, Gulf of Panama) across a well-documented upwelling gradient [e.g. 73] along 1400 km of coastline in Panama (Figure 1). Four reefs (Uva Island, Granito de Oro, Canal de Afuera Island and Bahia de Damas on Coiba Island) were sampled in the Gulf of Chiriqui in the west, where upwelling is absent and sea surface temperatures rarely drop below 25°C [73]. Coastal coral reefs are poorly developed in the Gulf of Chiriqui due to extensive freshwater runoff, but substantial reef development has occurred around its numerous islands, including the largest (136 ha), thickest (13 m) and oldest (5600a) TEP reefs [30], [73]. Two reefs (Achotines Bay and Iguana Island) were sampled in the centrally located Azuero Peninsula where occasional moderate upwelling occurs [74]. Coastal reefs are more common in the Azuero Peninsula than in the gulfs, most likely due to reduced freshwater runoff. Three reefs (Saboga and Contadora Islands in the Perlas Archipelago and Taboga Island) were sampled in the eastern Gulf of Panama where strong seasonal upwelling leads to declines in sea surface temperature to ∼15°C [73]. Coastal reefs are uncommon in the Gulf of Panama and reef development occurs primarily around near-shore islands and in the Perlas Archipelago.

### Sampling and Genetic analyses

On each reef, 18 to 36 nubbins (2 cm) of *P. damicornis* were sampled every ten meters along reef sites composed of dense stands of *P. damicornis* to analyze its small-scale spatial genetic structure. Samples were preserved in Guanidinium-Isothiocyanate (GITC) DNA buffer for genetic analysis [126]. DNA was extracted using standard Phenol-Chloroform extraction and ethanol precipitation.

Ten microsatellite loci were amplified using primers developed by Magalon *et al.* [127] (Pv2 & Pv6) and Starger *et al.* [128] (Pd2, Pd4, Pd5, Pd6, Pd7, Pd8, Pd9 & Pd10). Six of these loci (Pd2, Pd4, Pd5, Pd6, Pv2 & Pv6) amplified consistently using modified PCR protocols. PCR amplifications were carried out separately for each locus, following a nested PCR protocol for labeling, including forward primers with M13 tails and M13 primers with IR-labels [129]. Each PCR mix contained 2 pmol forward and 8 pmol of each reverse and m13 primer in a 10 µl reaction with 1 µl 10× PCR buffer, 0.2 mM dNTPs, 0.25U AmpliTaq DNA polymerase (Applied Biosystems) and 0.5 µl template DNA. PCR profiles varied slightly by locus: For Pd2, Pd4, Pd5 and Pd6, the PCR profile consisted of 30 cycles at 94°C for 45 s, 55°C for 35 s and 72°C for 35 s followed by 8 cycles with a lower annealing temperature (53°C) for the M13 labeling. For Pv2 and Pv6, the profile was modified to 30 cycles at 94°C for 45 s, 56°C for 45 s and 72°C for 30 s followed by 8 labeling cycles with 53°C annealing temperature. Both protocols included an initial 3 min denaturing step at 94°C and a final extension step at 72°C for 7 min. The PCR products with incorporated infrared dyes were then sized and scored on a LICOR 4300 Genetic Analyzer.

### Population genetics

Population genetic statistics, including allele frequencies, clonality, probability of identity, F-statistics and Analyses of Molecular Variance (AMOVA) were calculated using GenAlEx 6.1 [130]. The program Micro-Checker 2.3 [131] was used to test for stutter bands, large allele dropout and null alleles. Linkage disequilibrium among loci was assessed using a Markov chain method [132] in GENEPOP 4.0.10 [133], followed by sequential Bonferroni correction to account for multiple comparisons [134]. Clones were identified in GenAlEx and because they introduce biases into population genetic analyses, only unique genets were used unless otherwise noted.

Population genetic structure was calculated as F_ST_ [135], which assumes an infinite allele model [136], and R_ST_ [137], which is based on a stepwise mutation model of allele evolution [138]. Hierarchical AMOVA were used to estimate genetic structure among regions, among populations and among populations within regions [139]. Statistical significance of R_ST_ and F_ST_ is based on 999 permutations. R_ST_ recovered more genetic structure, especially among regions, whereas F_ST_ had a lower resolution and higher intra-regional variation (F_RT_ = 15% of F_ST_ compared to R_RT_ = 50% of R_ST_). As a result, we preferentially focus on R_ST_ (e.g. Tables 3 & 4). Principal Coordinate Analyses [140], implemented in GenAlEx, were used to detect and visualize the major patterns of pairwise R_ST_ population comparisons. Isolation by distance among populations was tested using a Mantel test, implemented in GenAlEx 6.1, to test for an association between pairwise population genetic differences and geographic distances.

The Bayesian clustering method implemented in STRUCTURE 2.3 [141] was used to infer the likely number of genetic clusters (K) in the dataset. STRUCTURE estimates the posterior probability P (X | K) that the data fit the hypothesis of K clusters using a Markov chain Monte Carlo approach to optimize genotypic equilibrium (linkage equilibrium and HWE) within each cluster. Five independent runs for each K from 2 to 19 were conducted using the admixture model and independent allele frequencies with a burn-in period of 10^5^ steps and 10^6^ Markov chain Monte Carlo replications for data collection (Figure S1). STRUCTURE runs were aligned using Clumpp1.1 [142] and the bar plot was generated with Distruct1.1 [143]. To verify the optimal value for K, we compared the P (X | K) results with ΔK, the second order rate of change in the likelihood of K [144], which corresponds to the strength of genetic subdivision among clusters in the data (Figure S1b).

Fine-scale Spatial Genetic Structure (SGS) within populations was analyzed using the program SPAGeDi 1.2 [145]. To distinguish between spatial genetic structure (SGS) due to clonal aggregations and SGS among unique genets, we analyzed and compared two different dataset: one dataset consisted of all 207 samples including clones (i.e. the ramet dataset) and one dataset contained only the 194 unique genotypes excluding clones (i.e. the genet dataset). SGS was estimated for each of the three regions separately and for all populations combined using the pairwise kinship coefficient F_ij_ [78], [146]. Other kinship coefficients including Ritland [147] and Moran's *I* [108] produced similar results (not shown). 95% confidence intervals and standard errors were estimated by 10,000 permutations of the genetic and the spatial datasets. Kinship values outside the 95% confidence intervals were interpreted as significant SGS at the spatial distance. Three parameters were used to describe and compare SGS among regions and studies: F_(1)_, genetic patch size and Sp. F_(1)_ is the average kinship among individuals within the smallest distance interval (10 m). Its statistical significance (p) was obtained by comparing the slope b(F) of F(r) on ln(r) to 9999 random permutations of individuals among locations within populations using a Mantel test. The genetic patch size is the distance that corresponds to the first x-intercept of the kinship correlogram [cf. 148]. Within the genetic patch, individuals are more closely related than the population average, i.e. the association between genotypes is positive, while individuals outside of the patch are genetically independent, i.e. their correlation is negative. The Sp statistic (Vekemans and Hardy [78] is based on F_(1)_ and the decrease of SGS with distance (b_(F)_).

## Supporting Information

Figure S1 **Results of the Bayesian clustering approach implemented in STRUCTURE 2.3** [**141**] **that were used to infer the number of genetic clusters (K) in the microsatellite dataset.** The graph shows the mean log likelihood L(K) for K = 1–19 (±SD) and ΔK, the second order rate of change of L(K) for K = 2–18 [144]. The most likely number of cluster is K = 5, since it has the highest likelihood value (L(K)  = −2059) and a local maximum for ΔK.
(EPS)
